# Trait and State Differences in OCD Patients with Comorbid Mood Disorders: A Comparative Analysis of Demographic and Clinical Scales

**DOI:** 10.1192/j.eurpsy.2025.1715

**Published:** 2025-08-26

**Authors:** S. Y. Lee, C. H. K. Park

**Affiliations:** 1 Asan medical center, Seoul, Korea, Republic Of

## Abstract

**Introduction:**

Obsessive-compulsive disorder (OCD) is a chronic condition that frequently co-occurs with mood disorders such as major depressive disorder (MDD) and bipolar disorder (BD), complicating both prognosis and treatment. The presence of MDD or BD in OCD patients is associated with more complex clinical presentations and worse outcomes.

**Objectives:**

Despite the high prevalence of these comorbidities, few studies have thoroughly compared the traits and states of OCD patients with comorbid mood disorders. This study aims to explore the differences in traits and states among OCD patients with comorbid mood disorders, including MDD, bipolar disorder I (BD1), and bipolar disorder II (BD2).

**Methods:**

The study included 114 OCD patients: 21 without mood disorders, 32 with MDD, 47 with BD2, and 14 with BD1. Demographic variables such as family history of psychiatric disorders and history of pharmacological treatment for OCD were analyzed. Participants were evaluated using standardized tools such as the Patient Health Questionnaire-9 (PHQ-9), Generalized Anxiety Disorder-7 (GAD-7), Temperament Evaluation of Memphis, Pisa, Paris, and San Diego Autoquestionnaire (TEMPS-A), Obsessive-Compulsive Inventory (OCI), Depressive Symptom Inventory Suicidality Subscale (DSI-SS). Statistical analyses including one-way analysis of variance (ANOVA) and chi-squared tests were conducted to identify significant differences between groups.

**Results:**

Patients with comorbid bipolar disorder (BD), particularly those with BD1, had a significantly higher prevalence of psychiatric family history (85.7%, p = .031). Pharmacological treatment for OCD was less frequent in patients with BD, with the lowest rate in the BD2 group (61.7%, p = .008). Compared to patients without mood disorders or those with MDD, OCD patients with BD showed higher scores in several temperament dimensions, including cyclothymic temperament (p < .001), depressive temperament (p = .007), hyperthymic temperament (p = .006), anxious temperament (p < .001), and irritable temperament (p < .001). These patients also exhibited more severe depressive symptoms (p < .001), higher anxiety levels (p < .001), and greater suicidality (p = .002). Obsessive-compulsive symptoms, particularly neutralizing behaviors (p = .010), ordering behaviors (p < .001), and hoarding behaviors (p < .001), were more pronounced in the BD groups.

**Conclusions:**

OCD patients with comorbid BD show distinct clinical profiles compared to those with MDD. They have a stronger genetic predisposition to psychiatric disorders and are less likely to receive pharmacological treatment for OCD. These patients also experience more severe depressive symptoms, anxiety, and obsessive-compulsive traits, complicating treatment. The findings highlight the need for comprehensive evaluations and personalized treatment plans for OCD patients with mood disorder comorbidities.

**Disclosure of Interest:**

None Declared

